# Screening and identification of MicroRNAs expressed in perirenal adipose tissue during rabbit growth

**DOI:** 10.1186/s12944-020-01219-5

**Published:** 2020-03-07

**Authors:** Guoze Wang, Guo Guo, Xueting Tian, Shenqiang Hu, Kun Du, Qinghai Zhang, Jingxin Mao, Xianbo Jia, Shiyi Chen, Jie Wang, Songjia Lai

**Affiliations:** 1 Farm Animal Genetic Resources Exploration and Innovation Key Laboratory of Sichuan Province, Sichuan Agricultural University, 211# Huimin Road, Wenjiang, 611130 Sichuan China; 2 Guizhou Medical University, Guiyang, 550025 China; 3 College of Pharmacy and Biological Engineering, Chengdu University, Chengdu, 610106 China; 4 Southwest University, Chongqing, 400715 China

**Keywords:** Rabbit, MicroRNA, Adipose tissue, MiRNA-seq, Lipid metabolism

## Abstract

**Background:**

MicroRNAs (miRNAs) regulate adipose tissue development, which are closely related to subcutaneous and intramuscular fat deposition and adipocyte differentiation. As an important economic and agricultural animal, rabbits have low adipose tissue deposition and are an ideal model to study adipose regulation. However, the miRNAs related to fat deposition during the growth and development of rabbits are poorly defined.

**Methods:**

In this study, miRNA-sequencing and bioinformatics analyses were used to profile the miRNAs in rabbit perirenal adipose tissue at 35, 85 and 120 days post-birth. Differentially expressed (DE) miRNAs between different stages were identified by DEseq in R. Target genes of DE miRNAs were predicted by TargetScan and miRanda. To explore the functions of identified miRNAs, Gene Ontology (GO) enrichment and Kyoto Encyclopedia of Genes and Genomes (KEGG) pathway analyses were performed.

**Results:**

Approximately 1.6 GB of data was obtained by miRNA-seq. A total of 987 miRNAs (780 known and 207 newly predicted) and 174 DE miRNAs were identified. The miRNAs ranged from 18 nt to 26 nt. GO enrichment and KEGG pathway analyses revealed that the target genes of the DE miRNAs were mainly involved in zinc ion binding, regulation of cell growth, MAPK signaling pathway, and other adipose hypertrophy-related pathways. Six DE miRNAs were randomly selected, and their expression profiles were validated by q-PCR.

**Conclusions:**

This is the first report of the miRNA profiles of adipose tissue during different growth stages of rabbits. Our data provide a theoretical reference for subsequent studies on rabbit genetics, breeding and the regulatory mechanisms of adipose development.

## Introduction

MicroRNAs (miRNAs) are endogenous non-coding RNAs, typically 18~26 nucleotides in length, that regulate gene expression in eukaryotic cells. Mature miRNAs are produced from long primary transcripts through a series of nucleases that are further assembled into RNA-induced silencing complexes. These complexes recognize target mRNAs by complementary base pairing, leading to mRNA degradation and the inhibition of translation [1]. MiRNAs regulate a wide range of physiological processes, including growth and development, virus defense, cell proliferation, apoptosis and fat metabolism. Adipose tissue controls metabolism through secretion of hormones, cytokines, proteins, and miRNAs that affect the function of cells and tissues throughout the body [2]. Meanwhile, it has been well documented that miRNAs regulate adipose tissue development, which are closely related to subcutaneous and intramuscular fat deposition [3, 4] and adipocyte differentiation [5]. MiRNAs, including miR-27b [6], miR-103 [7] and miR-148a [8] regulate adipogenic processes, promoting or inhibiting adipogenesis in animals. MiRNAs can be a new target for studying the molecular mechanisms governing fat development, growth and deposition in animals.

To-date, the role of miRNAs during fat development has been reported in humans, mice, livestock and poultry. Gu and colleagues [9] screened miRNAs in bovine adipose and breast tissues and identified 59 differentially expressed (DE) miRNAs, of which 5 differed from known mammalian miRNAs. Wang et al. [10] constructed an in vitro adipogenesis model of Crest-feather ducks identified 105 DE miRNAs by deep miRNA-sequeing, of which 12 were newly predicted and related to adipogenesis including miR-223, miR-184-3p and miR-10b-5. He et al. [11] also found that miR-148a-3p could be involved in regulating rabbit preadipocyte differentiation by inhibiting the expression of tensin homolog (*PTEN*).

As an important economic and agricultural animal, rabbits are the sources of meat and fur, and widely used as experimental models in biomedical research. In addition, the adipose tissue of rabbits has low deposition rates during growth, making it an ideal model to study adipose regulation [12–16]. However, studies on the miRNAs related to fat deposition during the growth and development of rabbits are limited. In this study, we performed miRNA-sequencing during three important stages of fat deposition (35, 85 and 120 days post-birth) of rabbits to identify key miRNAs that regulate adipose growth. Our findings provide a theoretical reference for subsequent studies on rabbit genetics and breeding and the regulatory mechanism of adipose development.

## Materials and methods

### Animal and sample collection

Given the plasticity and maturation of rabbit adipose tissue, Tianfu Black rabbits (an indigenous breed of Sichuan province in China) aged 35 (weaned stage), 85 (slaughter stage) and 120 (initial breeding stage) days were used in this study. All rabbits used were raised under the condition with the same diet (a commercial pelleted food (16% protein, 10.8 MJ/kg) after weaning) and environmental temperature and given free access to water and food. In this study, rabbits were euthanized by cervical dislocation. Three biological replicates of perirenal adipose tissue were collected for 35 days (YR) and 120 days (TR), and two for 85 days (MR). The samples were snap frozen in liquid nitrogen, and stored at − 80 °C until RNA extraction.

### Total RNA extraction

Total RNA was extracted from 50 to 60 mg of perirenal adipose tissue in each sample using the Trizol Reagent (Life Technologies, Carlsbad, CA, USA) according to the manufacturer’s instructions and dissolved in RNase free water. RNA purity and integrity were determined using a Nanodrop (Thermo Fisher Scientific, Waltham, MA, USA) and an Agilent Bioanalyzer 2100 system (Agilent Technologies, CA, USA), respectively. Then, RNA concentrations were measured using a Qubit^@^ RNA Assay Kit and Qubit^@^ 2.0 Fluorometer (Life Technologies, Carlsbad, CA, USA). Only samples with RNA Integrity scores > 8 were used for sequencing.

### MiRNA library construction and sequencing

MiRNA libraries were constructed and sequenced by Mega Genomics Co.,Ltd., (Beijing, China). Sequencing libraries were prepared using TruSeq Small RNA Sample Prep Kits (Illumina, San Diego, USA) according to the manufacturer’s instructions. Briefly, 3′ and 5′ linkers were used for cDNA synthesis, and PCR amplification. Target fragments were gel-purified and the quality of the libraries was assessed using Bioanalyzer 2100 (Agilent, CA, USA). Libraries were sequenced on an Illumina Hiseq 2500 platform and 50-bp single-end reads were generated.

### MiRNA bioinformatics analysis

MiRNAs were analyzed using ACGT101-miR (LC Sciences, Houston, Texas, USA). The analysis procedure was as follows: (1) 3′ connector and non-specific sequences were removed to obtain clean data; (2) the length of the sequences were maintained at 18~26 nt through length screening; (3) mRNAs, RFam and Repbase databases were used for comparative analysis and the filtration of remaining sequences; (4) fitering was used to obtain effective data and precursors were compared to rabbit reference genomes (GCF_000003625.3_OryCun2.0_genomic.fa) for miRNA identification; (5) differentially expressed (DE) miRNAs were analyzed with *p*-value (FDR) ≤ 0.05 as the threshold; (6) target genes of DE miRNAs were predicted by TargetScan [17–19] and miRanda [20, 21]; (7) GO functional annotation and KEGG pathway analysis were used to investigate the functional enrichment of the identified miRNA target genes with *p*-value ≤0.05 as the criteria.

### Validation of DE miRNA by q-PCR

Primers for the miRNAs and internal controls (Additional file 1) were designed using Primer-BLAST (https://www.ncbi.nlm.nih.gov/tools/primer-blast/). MiRNA-specific primers were synthesized by Sangon Biotech Co. Ltd. (Shanghai). Six DE miRNAs were reverse transcribed into cDNA using Mir-X™ miRNA First-Strand Synthesis Kit (Takara, Dalian, China) according to the manufacturer’s protocol. Q-PCR was performed using SYBR® Green II qRT-PCR kit (Takara, Dalian, China) according to the manufacturer’s instructions. A total 10 μl of PCR mixture contained of 4.5 μl SYBR® Green II, 1 μl cDNA, 0.5 μl of 10 μM forward and reverse primers and 3.5 μl RNase free dH_2_O. PCR was performed on a Rotor gene 6000 PCR System (QIAGEN, Hiden, Germany) as follows: 95 °C for 30 s, followed by 40 cycles at 95 °C for 5 s, and 61 °C for 20 s. The expression levels of the miRNAs were normalized to U6 small nuclear RNA. Relative miRNA expression was calculated using the 2^-ΔΔCt^ method [22]. Data were expressed as the mean ± standard error of the mean (SEM).

### Statistical analysis

T-test was used to analyze the DE miRNAs. Statistical analysis was performed using SPSS Statistics 20.0 (SPSS Inc., Chicago, IL, USA). The *p*-value < 0.05 was considered statistically significant.

## Results

### Overview of MiRNA sequencing

Eight miRNA libraries of YR-1, YR-2, YR-3, MR-1, MR-2, TR-1, TR-2 and TR-3 were constructed and divided into YR, MR and TR groups. Up to 1.6 GB of data was obtained, and 8 libraries consisting of raw reads ranging from 10138426 to 15721988 were generated. FastQC (0.10.1) software was used to control data quality through the removal of 3ADT & length filters (80% A or C / G or T; 3 N; A alone; C without G; T alone; G alone; T without A; C alone; or continuous nucleotide dimers and trimers) and junk reads. After filtering and comparison to cellular mRNAs, RFam and Repbase databases, 1416639 ~ 14139070 valid reads were obtained. The number of effective unique copies obtained from the libraries were 172905~381169, accounting for 29.91%~ 47.15% of the total sample (Additional file 2).

### Length distribution of the candidate miRNAs

**Fig. 1:**
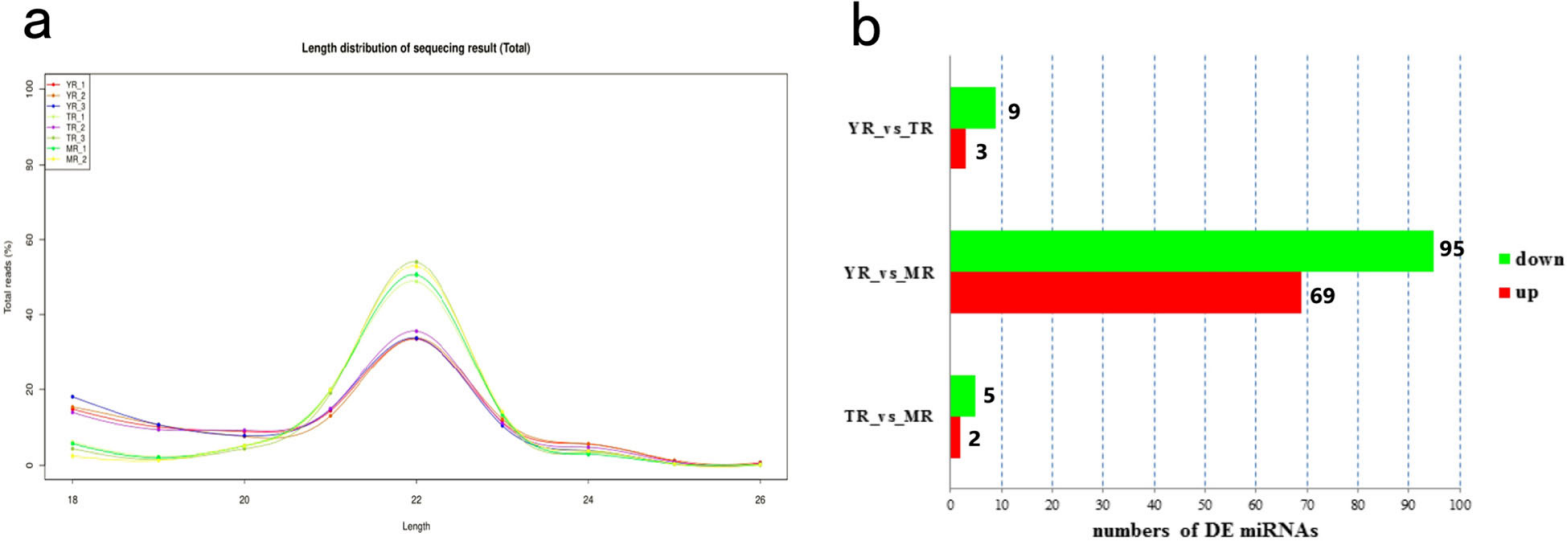
Identification of rabbit miRNAs. **a**. Length distribution of the 987 miRNAs. **b**. Up and down-regulated miRNAs in the rabbit perirenal adipose during the three growth periods (*P* < 0.05)

Following counting and analysis of the original sequencing data, the length distribution of the miRNAs in the 8 libraries was similar, varying from 18 nt~ 26 nt, with 22 nt miRNAs most frequent (Fig. 1a). To further analyze the validity of the sequencing data, statistical analysis on the length distribution of unique miRNAs was performed on filtered datasets. The results showed that the number of the miRNAs in the 8 libraries were similar to > 60% of the reads, which were of 20~24 nt in size, consistent with the characteristics of Dicer enzyme cleavage. Some miRNAs were in 25 nt and 26 nt in length, accounting for < 6% of the total sequences (Additional file 3).

### Annotation and identification of miRNAs

**Fig. 2:**
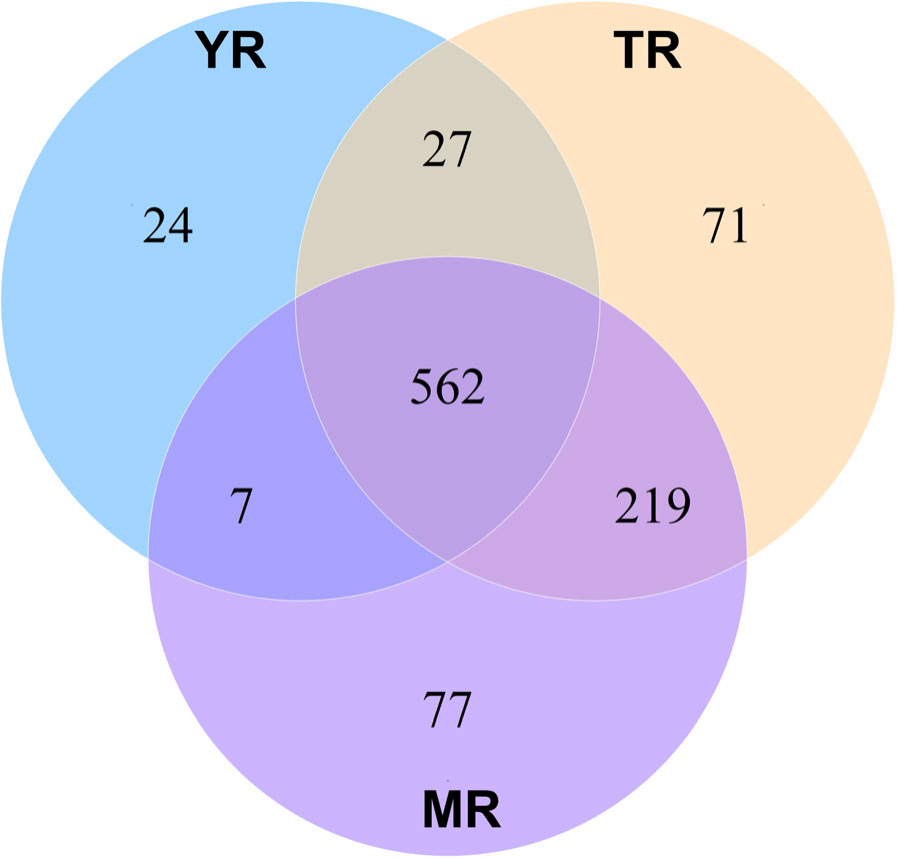
A Venn diagram showing the miRNAs at the three growth periods

To obtain conserved miRNAs in rabbit adipose tissue, the ACGT101-miR (4.2) tool was used to compare the reference genome-matched reads with the known mature miRNAs in the miRase database. As a result, a total of 987 miRNAs (Additional file 6) were obtained during the three adipose growth stages, including 780 known miRNAs and 207 newly predicted miRNAs. Meanwhile, 131 miRNAs were highly expressed, 652 were moderately expressed, and 204 were expressed to low-levels. In addition, miRNA expression varied during different adipose growth stages 620 miRNAs obtained by YR (35 days), 865 obtained by MR (85 days), and 879 obtained by TR (120 days). A Venn diagram was constructed using total miRNAs showed that 562 miRNAs were common in all three growth stages (Fig. 2). These results showed that miRNA expression gradually increases during the adipose growth of rabbits.

The length distribution of the 987 miRNAs was assessed. The results showed that the lengths ranged from 18 to 26 nt with 398 miRNAs of 22 nt in length, accounting for the highest proportion (40.32%). while 26 nt miRNAs were least common (0.61%). The length distribution of the 780 known miRNAs was consistent with the total miRNAs, with the majority 22 nt in length (43.08%). Of the 207 newly predicted miRNAs, none were 26 nt, 2 were 25 nt and 62 were 22 nt in length (Additional file 4).

The 987 miRNAs were next analyzed to assess their evolutionary conservation. The results showed that miRNAs originated from 103 families and the numbers were differentially distributed. Members of the let-7 and miR-10 families were most frequent (11 miRNAs). Single miRNAs were identified for miR-196, miR-130 and miR-205 families.

### Identification of differentially expressed miRNAs

The DEGseq package in R was used to identify DE miRNAs and adjusted *p*-value (FDR) ≤ 0.05 was taken as standard to screen DE miRNAs during the three stages of rabbit adipose growth. A total of 174 DE miRNAs were obtained from 987 miRNAs in the three groups, of which 70 were up-regulated and 104 were down-regulated (Additional file 5), indicating that the proportion of down-regulated miRNAs during rabbit adipose growth was significantly higher than the number of up-regulated miRNAs. Pairwise comparisons of the YR, MR and TR miRNA data showed 7, 164, 12 DE miRNAs between the respective growth stages (Additional file 5). Among these, the number of DE miRNAs in the YR-vs-MR comparison group was the highest (Fig. 1b). Through in-depth analysis of the miRNA data obtained from inter-group comparisons, 12 DE miRNAs of YR-vs-TR showed middle expression (10 < FPKM < mean of total miRNAs expression), 3 DE miRNAs of TR-vs-MR showed middle expression, and 49 DE miRNAs showed high expression (FPKM ≥ mean of total miRNAs expression) in YR-vs-MR, indicating that miRNA expression was more active at 85 days of rabbit adipose growth.

**Fig. 3:**
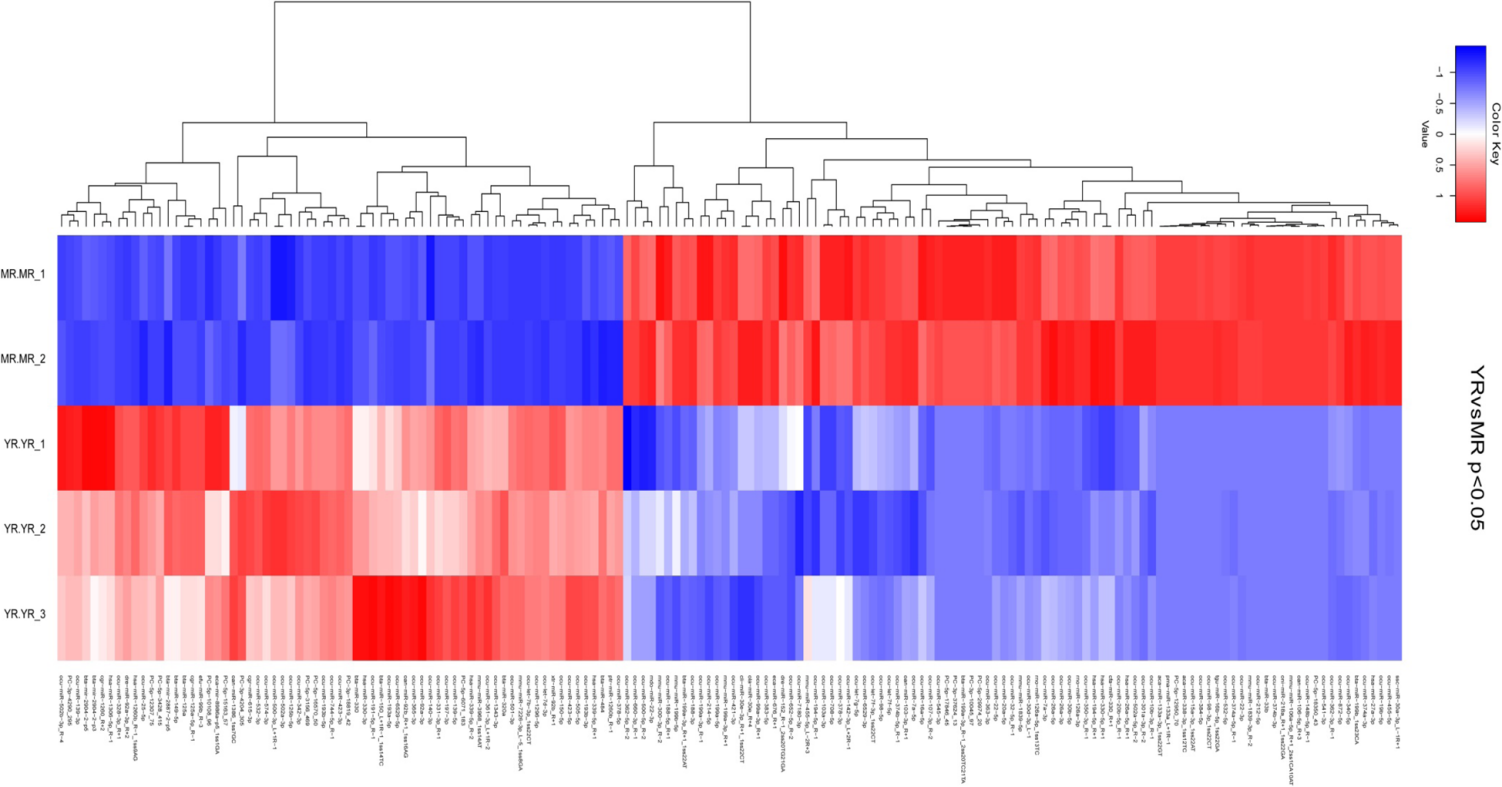
Hierarchical clustering analysis of the miRNA expression profiles from YR-vs-MR with 164 DE miRNAs. The abscissa represents the type of miRNAs, and the ordinate represents the sample. Data are expressed as FPKM. Red - relatively high expression and Green - relatively low expression

To intuitively understand the expression of DE miRNAs in YR-vs-MR, hierarchical clustering was performed on the 164 screened miRNAs (Fig. 3). As shown in Fig. 3, 164 miRNAs showed differential expression patterns according to the different growth stages, and the libraries of each group were comparable. The number of highly expressed DE miRNAs (red) in the MR group was significantly higher than the YR group.

### Enrichment analysis of the target genes of DE miRNAs

**Fig. 4:**
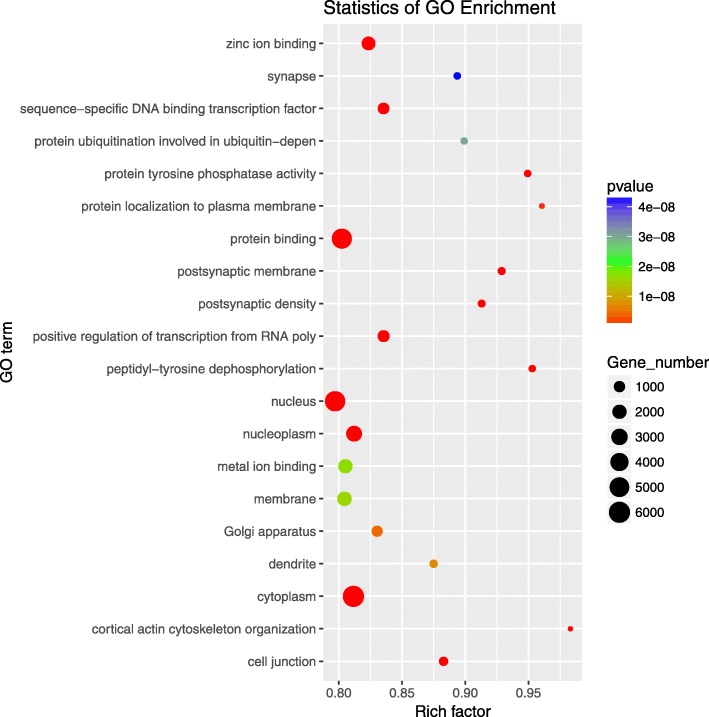
Top 20 significant terms of GO enrichment analysis of target genes of DE miRNAs at *p*-value < 0.05

Target genes of the DE miRNAs were predicted using TargetScan and miRanda software, and their intersections were taken as final target genes. The number of targets of the 174 DE miRNAs was 13,204. According to the relationship between miRNAs and their target genes, the GO enrichment analysis showed that 13,347 GO terms were obtained, including 8807 terms of biological process (BP), 1279 terms of cell component (CC), and 3261 terms of molecular function (MF). Among these, 1048 terms were significantly enriched (*P*<0.05). Analysis of the 1048 GO terms showed that the target genes of DE miRNAs were significantly enriched in protein binding, cytoplasm, zinc ion binding, regulation of cell growth, and ATP binding (Fig. 4).

**Fig. 5:**
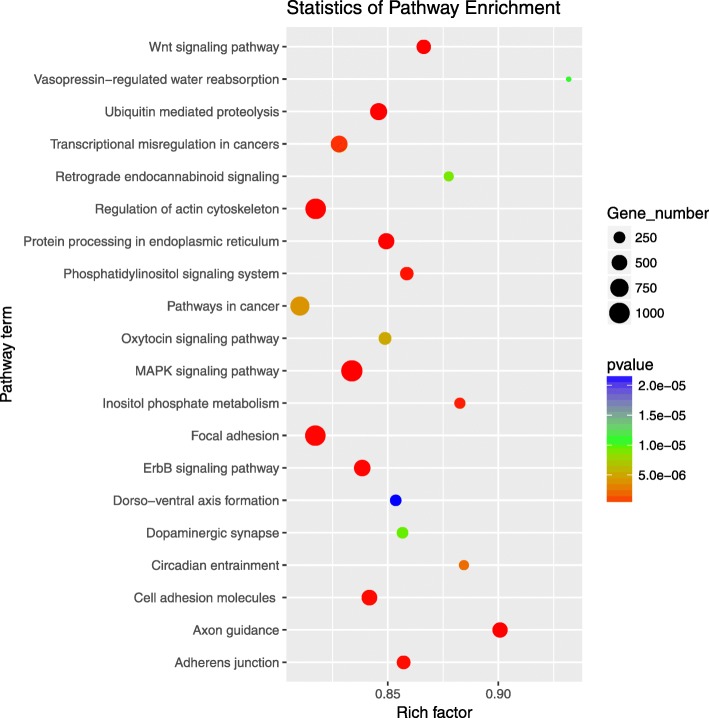
The top 20 significant terms of KEGG Pathway analysis of target genes of DE miRNAs at *p*-value < 0.05

To more comprehensively describe the functions of the target genes during the different growth stages, enrichment analysis of the KEGG pathways was used to understand the biological functions of the genes. The results found that the target genes of DE miRNAs were enriched in 315 KEGG pathways, 91 of which were significantly enriched (*P*<0.05), including the MAPK signaling pathway, Wnt signaling pathway, Renin secretion, FoxO signaling pathway, and Aldosterone synthesis and secretion (Fig. 5).

### Validation of DE miRNAs

**Fig. 6:**
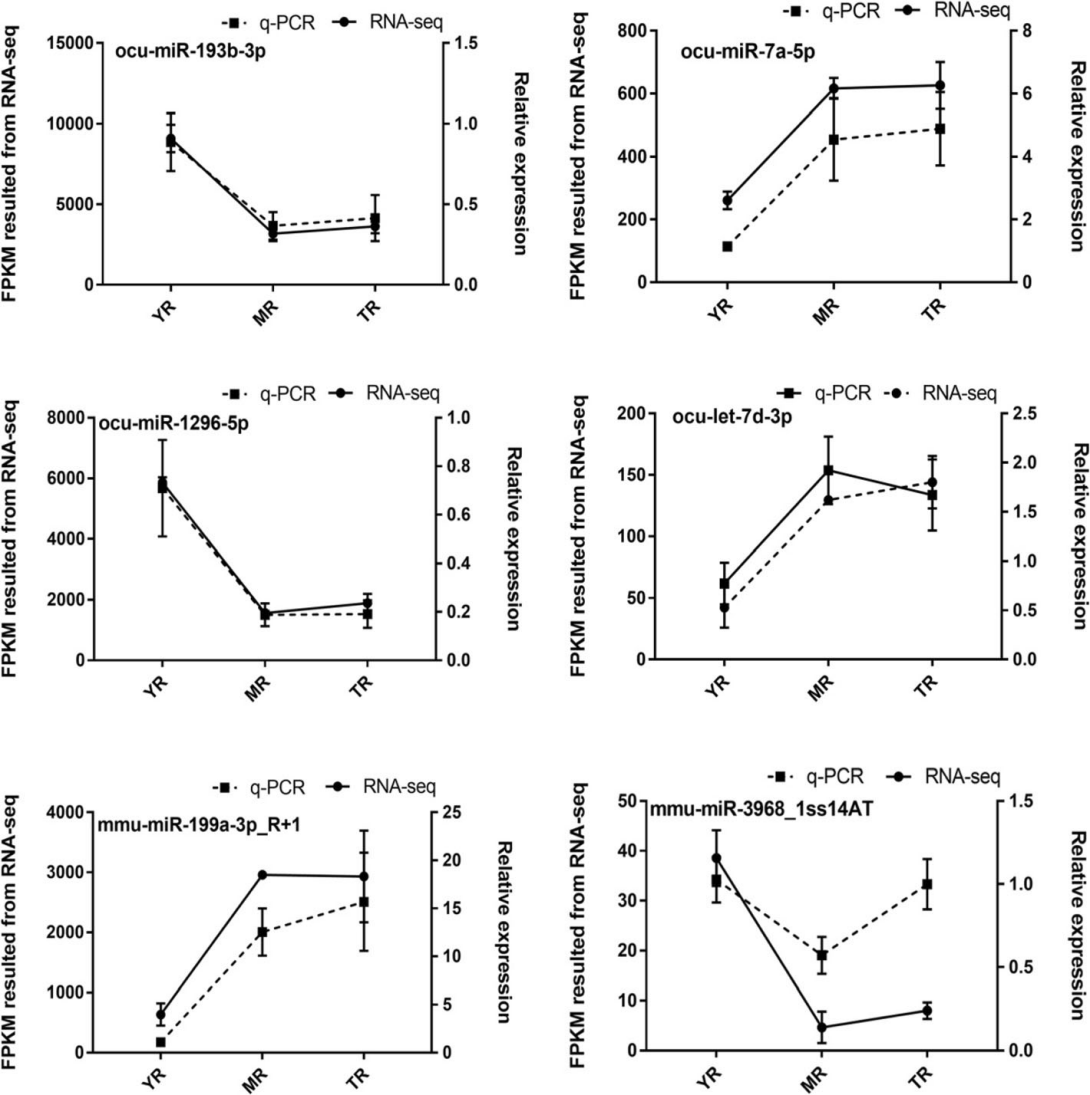
Validation of the six randomly selected DE miRNAs by q-PCR

To validate the reliability of the miRNA-seq data, six miRNAs (ocu-miR-1296-5p, ocu-miR-193b-3p, mmu-miR-3968_1ss14AT, mmu-miR-199a-3p_R + 1, ocu-let-7d-3p and ocu-miR-7a-5p) were randomly selected from 174 DE miRNAs to validate their expression profiles at three growth stages by q-PCR. The results showed that all six miRNAs were differentially expressed during the different growth stages (*p*-value < 0.05). In addition, the six miRNAs exhibited a similar trend between the results of miRNA-seq and q-PCR (Fig. 6). Therefore, the FPKM obtained from the miRNA-seq datasets can be reliably used to determine miRNA expression and confirmed the importance of DE miRNAs during the growth of rabbit adipose tissue.

## Discussion

In eukaryotes, miRNAs act as a broad class of widely occurring small-molecule ncRNAs that regulate gene expression though targeting mRNA transcription degradation and translation [23, 24]. MiRNAs play important roles in animal growth and development, host immune responses, adipose differentiation and lipid metabolism. Currently, approximately 2000 miRNAs are recognized in human and mouse genomes, and the majority of which are expressed in a tissue-dependent manner [25, 26]. However, studies on the regulation of miRNAs during rabbit adipose growth and development are lacking. Here, we used miRNA-sequencing to identify 987 miRNAs during three important stages of rabbit adipose growth, including 780 known miRNAs and 207 newly predicted miRNAs. The miRNAs were derived from 103 families with 643 seed region specificities, including miR-30 and miR-204. Studies have shown [27] that miR-30a and miR-30d induce lipogenesis in obese patients through targeting *RUNX2* and miR-30c respectively, promoting the differentiation of human adipocytes [28].

We compared the identified miRNAs to other species, which were distributed into 67 miRNAs that included has, mmu, and bta. In-depth analysis of the obtained miRNAs lengths revealed that both known miRNAs and newly predicted miRNAs were mainly of 22 nt in length, and increased in abundance during the three growth stages. Similarly, using HiSeq sequencing, Wang and colleagues [29] identified 329 known miRNAs and 157 new miRNAs during the development of porcine adipose. Additionally, Wang and coworkers [10] identified 105 DE miRNAs through the deep sequencing of duck adipose tissue and differentiated preadipocytes in vitro and demonstrated that miRNA expression varies among different species.

In the present study, the DEGseq R language package was used to identify DE miRNAs. We identified 174 DE miRNAs during the three growth stages of rabbits that were mostly down-regulated. A comparison of each of the stages showed that the number of DE miRNAs at 35 days and 85 days was highest. Adipose growth in the rabbits was significantly affected by age and miRNA expression was more prevalent during early growth stages. Of the 174 DE miRNAs, some were distributed in miR-133, miR-30 and let-7 families. A similar study showed that miR-133a was expressed in brown and white adipose tissue, directly targeting the 3’UTR region of *Prdm16* [30]*.* miR-let-7b regulates the level of human adipose tissue-derived mesenchymal stem cells (hAT-MSCs), and the transient inhibition of miR-let-7b enhances the differentiation of hAT-MSCs [31]. These results suggest that the DE miRNAs identified in this study play regulatory roles during adipose growth in rabbits.

MiRNAs pair with the 3’UTRs of target genes to inhibit translation and silence gene expression at the post-transcriptional level. It has been reported that 30–80% of the mammalian miRNAs target multiple cellular mRNAs [18]. In general, target genes regulated by the same miRNA originate from the same gene family [32]. In this study, the 174 DE miRNAs were predicted to target 13,204 genes with an average of 76 genes targeted for each predicted miRNA. Furthermore, the target genes regulated by single miRNAs originated from the same family, and the DE miRNAs showed obvious temporal characteristics.

Compared to lncRNAs [33], miRNAs and the target genes of DE miRNAs were mainly involved in GO functional terms including metabolic process, cell process and single organism process in the classification of biological processes, partial cells and organisms in the classification of cell components, and binding and catalytic activity in the classification of molecular functions. Based on our in-depth analysis of the 1048 significantly enriched GO terms, among the top 10 GO terms of biological processes, cell composition and molecular function, some terms that strongly promote growth and volume increase in adipocytes, including protein localization to the plasma membrane and protein ubiquitination involved in ubiquitin-dependent protein catabolic process, regulation of cell growth, cytoplasm, Golgi apparatus, membrane, protein binding, protein tyrosine phosphatase, zinc ion binding, ATP binding, and cadherin binding were identified. However, there were few related terms regarding glyceric acid absorption and lipid droplet formation during adipose hypertrophy. Recent studies on miRNA expression in human adipose tissue found that the expression of miRNAs was specific to the site of adipose tissue [34, 35]. Some miRNAs were associated with adipose tissue morphology, adipocyte size, and metabolic functions (fasting glucose and triglyceride). Combined with our data, the target genes of DE miRNAs more highly influenced cell membrane growth, protein synthesis and utilization, energy utilization and transformation, but their role in lipid droplet accumulation in adipocytes was not obvious.

Among 91 pathways, which were significantly enriched by KEGG, MAPK signaling pathway, Wnt signaling pathway and aldosterone synthesis and secretion pathways had been shown to regulate the growth and development of adipocytes. Related studies have shown that some miRNAs participate in the regulation of adipose deposition. For example, miR-148a promotes adipose synthesis by inhibiting the expression of *Wnt1* [8], while the over-expression of miR-10b L20 significantly increases the level of adipose and triglyceride [36]. In addition, miRNA families such as let-7, miR-30, miR-17, miR-148 [37] and miR-24 [38, 39] are involved in the adipose deposition of animals. miR-20a regulates adipocyte differentiation by targeting lysine-specific demethylase 6 b and transforming growth cytokine β signal [40]. Previous studies [41] assessed the anti-adipogenesis characteristics of miR-27b, which was down-regulated during adipocyte differentiation and weakened the induction of *PPARc.* The expression of miR-95 significantly correlated with adipocyte size, and its expression significantly increased during adipocyte differentiation [34]. Therefore, the results of this study suggest that miRNAs with tissue and developmental stage specificity play key roles in the growth and maturation of rabbit adipose tissue.

## Conclusions

To the best of our knowledge, this is the first report to perform miRNA profiling of rabbit perirenal adipose tissue during different growth stages, which identified 987 miRNAs and 174 DE miRNAs associated with adipogenetic pathways. These included the regulation of cell growth, zinc ion binding, MAPK signaling pathway, and Wnt signaling pathway. Therefore, these DE miRNAs regulate the growth and hypertrophy of adipose tissue in rabbits.

## Supplementary information

**Additional file 1.** Primer information of 6 MiRNAs used for q-PCR validation.

**Additional file 2.** Summary and quality assessments of the sequencing data.

**Additional file 3.** Sequence distribution of each unique MiRNA from each sample.

**Additional file 4.** Length distribution of the identified MiRNAs.

**Additional file 5.** The DE miRNAs of YR-vs-TR, YR-vs-MR and TR-vs-MR.

**Additional file 6.** The list of all expressed miRNAs.

## Data Availability

All data generated or analyzed during this study are included in this published article.

## Abbreviations

BP: Biological process
CC: Cellular component
DE: Differentially expressed
FPKM: Fragments per kilobase of transcript per million fragments mapped
GO: Gene ontology
KEGG: Kyoto Encyclopedia of Genes and Genomes
MF: Molecular function
MicroRNAs: MiRNAs
PTEN: Tensin homolog
